# Graph neural pre-training based drug-target affinity prediction

**DOI:** 10.3389/fgene.2024.1452339

**Published:** 2024-09-16

**Authors:** Qing Ye, Yaxin Sun

**Affiliations:** ^1^ School of Data Science and Artificial Intelligence, Wenzhou University of Technology, Wenzhou, China; ^2^ School of Computer Science and Technology (School of Artificial Intelligence), Zhejiang Normal University, Jinhua, China; ^3^ Zhejiang Aerospace Hengjia Data Technology Co. Ltd., Jiaxing, China

**Keywords:** drug-target affinity, pre-training model, graph isomorphism network, deep neural network, feature extraction

## Abstract

Computational drug-target affinity prediction has the potential to accelerate drug discovery. Currently, pre-training models have achieved significant success in various fields due to their ability to train the model using vast amounts of unlabeled data. However, given the scarcity of drug-target interaction data, pre-training models can only be trained separately on drug and target data, resulting in features that are insufficient for drug-target affinity prediction. To address this issue, in this paper, we design a graph neural pre-training-based drug-target affinity prediction method (GNPDTA). This approach comprises three stages. In the first stage, two pre-training models are utilized to extract low-level features from drug atom graphs and target residue graphs, leveraging a large number of unlabeled training samples. In the second stage, two 2D convolutional neural networks are employed to combine the extracted drug atom features and target residue features into high-level representations of drugs and targets. Finally, in the third stage, a predictor is used to predict the drug-target affinity. This approach fully utilizes both unlabeled and labeled training samples, enhancing the effectiveness of pre-training models for drug-target affinity prediction. In our experiments, GNPDTA outperforms other deep learning methods, validating the efficacy of our approach.

## 1 Introduction

Predicting drug-target affinity (DTAP) is a crucial research topic in drug development, which can be. used for discovering drug on-target and off-target effects. However, due to the increasing number of targets, it is difficult to fully validate the drug-target affinity (DTA) of drugs using biochemical experiments. In recent years, with the development of artificial intelligence technology, the use of computational methods for preliminary prediction of DTA has become an economically effective method. Graph neural networks (GNN) can extract features from graph-structured data and have been widely used in DTA, as drugs and targets are typically graph-structured data.

These methods can be divided into four categories. Firstly, numerous graphs have been created for drugs and targets, such as RDKit tool graph (Lin, 2020), sequence-predicted 2D contact maps (You and Shen, 2022), residue protein contact map (Jiang et al., 2020), R-radius subgraph (Tsubaki et al., 2019), weighted protein graphs (Jiang et al., 2022), which Secondly, numerous attention mechanisms have been incorporated into the GNN, such as the distance-aware molecule graph attention (Zhou et al., 2020), triplet-attention (Liao et al., 2022), atom aggregated graph (Lin, 2020), self-attention (Zhang et al., 2021; Liao et al., 2022), layer attention (Tang et al., 2023) and dynamically allocate attention (Meng et al., 2024). Thirdly, various deep learning frameworks have been devised for GNN-based DTAP, such as super-deep GNN (Yang et al., 2022), structure-aware interactive GNN (Li Shuangli et al., 2021), graph within GNN (Nguyen et al., 2022), hierarchical GNN (Chu et al., 2022), multiple output GNN (Ye et al., 2021), Data augmentation and feature fusion GNN (Fang et al., 2023), super edge GNN (Gu et al., 2023), diffusion GNN (Zhu et al., 2023), Molecular graphs and binding pocket graphs GNN (Wu et al., 2024), graph dilated convolution networks (Zhang et al., 2024), Bi-directional fusing intention network (Peng et al., 2024). Fourthly, GNNs have been utilized in conjunction with other network architectures for DTAP, such as recurrent neural network (Lin, 2020; You and Shen, 2022; Mukherjee et al., 2022), multi-subspace deep neural networks (Ye et al., 2023; Zhang Li et al., 2023).

Different graph can enhance understanding of atomic connectivity and residue interactions. The strengths of attention mechanisms lie in their ability to focus on relevant parts of the graph, enhancing the model’s representational power. Various deep learning frameworks offer diverse and innovative approaches for DTAP. Combining GNNs with other network architectures provides a comprehensive approach for DTAP, leveraging the strengths of different network types. Although the above deep neural networks based on GNNs can improve the performance of DTAP in various ways, most of the methods cannot address the issue of the scarcity of labeled training samples for DTA.

There have been some methods that have noticed this problem. A pre-trained language model based on bidirectional encoder representations from transformers is designed to extract semantic features of SMILES molecules (Qiu et al., 2024). Multiple Transformer-Encoder blocks were designed to capture and learn the proteomics, chemical, and pharmacological contexts (Monteiro et al., 2024). Transformer-based architecture was utilized to learn representation for drugs (Rafiei et al., 2024). GCN-BERT utilized two RoBERTa models to extract features for the drug and target (Lennox et al., 2021). CPCProt divided protein sequences into fxed-size segments and trained an autoregressor to distinguish subsequent segments of the same protein from random protein segments (Lu et al., 2020). SubMDTA proposed a self-supervised pretraining model based on substructure extraction and multi-scale features (Pan et al., 2023). ProtBert was utilized to extract the feature for the target (Zhang Xianfeng et al., 2023). Two modalities ProtBERT-BFD from ProtTrans2 and PSSM based descriptors are used to represent the target (Liyaqat et al., 2023). Four contrastive loss functions are considered to learn a more powerful model, such as Max-margin contrastive loss function, Triplet loss function, Multi-class N-pair Loss Objective, and NT-Xent loss function (Dehghan et al., 2024). These methods use pre-training to extract better features, but they fail to notice the significant difference between the pre-training objectives and samples, and the training objectives and samples for DTAP. Specifically, pre-training uses samples of drugs or targets individually, while DTAP utilizes samples of drug-target pairs for model training.

To overcome the beforementioned issues and further improve the DTAP performance of GNNs, this paper proposes a graph neural pre-training-based drug-target affinity (GNPDTA) prediction method. This approach divides the feature extraction for DTAP into two stages. In the first stage, a graph neural pre-training model is employed to extract low-level features of drugs and targets separately. During the process of drug-target affinity generation, we observe that drug-target affinity is generally related to their local fragments. Since target sequences tend to be longer, the pre-training model primarily extracts features of target fragments. Drug SMILES usually consist of 50 atoms, so the pre-training model focuses on extracting features of drug graph nodes. In the second stage, a convolutional neural network is utilized to combine the features of adjacent target fragments and drug graph nodes, resulting in features for predicting drug-target affinity.

GNPDTA has the following main contributions. One is that GNPDTA can Minimize discrepancy between pre-training and DTAP objectives. By devoting the initial stage exclusively to feature extraction, the proposed method effectively bridges the gap between pre-training objectives (which focus on individual drug or target samples) and DTAP objectives (which consider drug-target pairs). This ensures that the extracted features are highly pertinent to the DTAP task. Another is that The GNPDTA method introduces a two-stage approach tailored specifically for drug-target affinity prediction (DTAP). This strategy intelligently leverages distinct models and training methodologies at each stage, maximizing the utilization of both unlabeled and labeled data to enhance feature extraction effectiveness.

## 2 Materials and methods

### 2.1 Datasets

The GNPDTA method consists of two stages. In the first stage, a large amount of unlabeled data is used to train the pre-training model. For targets, the Swiss-Prot dataset (Swiss-Port dataset) is used for training the pre-training model, which includes 565,928 targets. For drugs, the CHEMBL dataset (Gaulton et al., 2017) is used for training the pre-training model, containing 2,105,464 drugs.

In the second stage, the proposed model was evaluated on five benchmark datasets of DTAP, namely, the Kiba (Tang et al., 2014), Davis (Davis et al., 2011), DTC (Tang et al., 2018), Metz (Metz et al., 2011), and Tox-Cast (US). The simple statistics for the sample information of these datasets are given in Table.1. It can be seen from Table.1 that there are only 2,111, 68, 5,983, 1,471, and 7,657 drugs and only 229, 442, 118, 170, and 328 targets on the above datasets. As a result, the prediction model could be hardly well trained only by these samples.

**TABLE 1:** Simple statistics for the sample information of five DTA datasets.

Data sets	Drugs	Targets	Used drug-targets pairs
Kiba	2,111	229	118,254
Davis	68	442	30,056
DTC	5,983	118	67,894
Metz	1,471	170	35,307
Tox-Cast	7,657	328	342,869

### 2.2 GNPDTA structure

**FIGURE 1:**
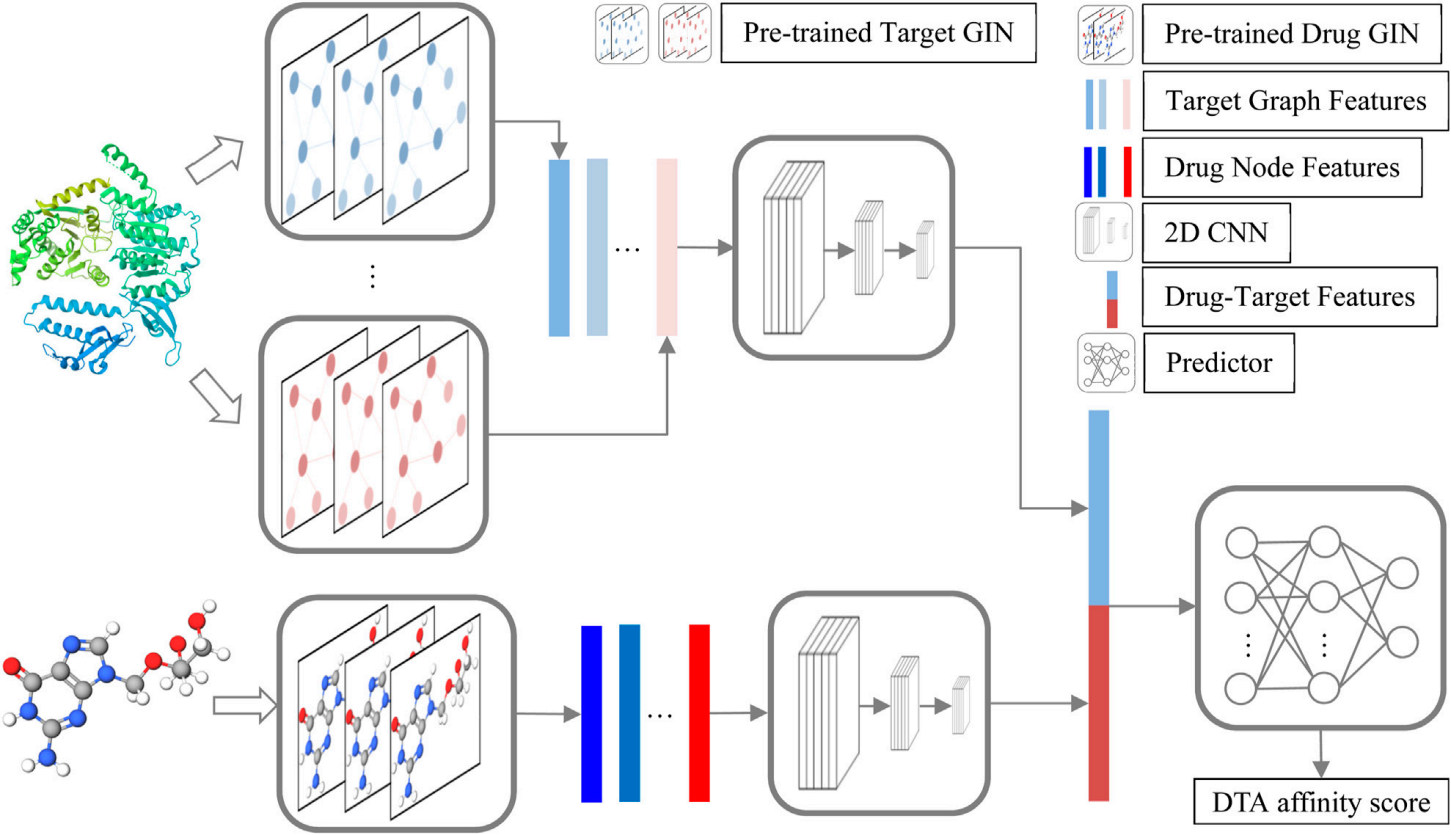
GNPDTA structure.

The structure of GNPDTA is depicted in Figure 1. As can be observed from Figure 1, GNPDTA comprises seven parts, including pre-trained target graph isomorphism network (GIN), pre-trained drug GIN, target graph features, drug node features, 2D convolutional neural networks (CNN) Layers, drug-target features, and predictor. The pre-trained target GIN and pre-trained drug GIN are trained using a large number of unlabeled target training samples and unlabeled drug training samples, respectively, to extract low-level features of targets and drugs. Target graph features are the low-level features of targets extracted by the pre-trained target GIN. Drug node features are the low-level features of drugs extracted by the pre-trained drug GIN. Two 2D CNNs are used to extract high-level features of drugs and targets from target graph features and drug node features, respectively. Drug-target features are obtained by concatenating target graph features and drug node features. Predictor utilizes Drug-Target Features to predict DTA.

### 2.3 Low-level features extracted by pre-trained GIN

Pre-training models can utilize a large number of unlabeled samples, which can be used to overcome the problem of insufficient drug-target affinity data. Furthermore, the drug and the target are two typical graph structure data, where they take atoms as nodes and bonds as edges. Therefore, two graph pre-training GINs are considered to extract drug and target features, where GIN is one of the types of GNN. GIN is chosen, for the reason that GIN’s complete graph convolution approach enables it to capture global features in graph structures, resulting in stronger expressive power when dealing with complex graph structures, where the drug and target are all complex graph structures.

Pushing different molecules away by contrastive approaches and randomly masking the discrete values and pre-training GNNs to predict them by masked atoms modeling are two most used graph pre-training models for molecules. Because drugs and targets are mainly composed of a few atoms such as C, H, O, N, or S, and then the ability to learn the drug and the target characteristics through masked atoms modeling (MAM) may be relatively weak. Therefore, an unsupervised graph-level representation learning via mutual information maximization (INFOGRAPH) (Sun et al., 2020) is used to learn the drug and the target representation, which belongs to a contrastive approach.

The objective of INFOGRAPH is to maximize the mutual information (MI) between the representations of entire graphs and the representations of substructures of different granularity (Sun et al., 2020).Given *N* training graphs $\left[g_{1},g_{2},\cdots ,g_{N}\right]\in G$ , the node features $h_{i}^{n}$ of *v*-th node of *g*_*i*_ can be defined as:

$$h_{\phi }^{n}=f_{g}\left(g^{n}\right)$$ (1)

where $f_{g}$ is a GIN, containing five graph convolutional layers.

The graph features of *g* can be defined as:

$$H_{\phi }\left(g\right)=\sigma \left(\frac{1}{N}\sum_{n=1}^{N}h_{\phi }^{n}\right)$$ (2)

where $\sigma \left(\cdot \right)$ is an activation function.

And then the mutual information between $h_{\phi }^{n}$ and $H_{\phi }\left(g\right)$ can be defined as:

$$\hat{\phi },\hat{\varphi }=\mathrm{arg max}\sum_{g\in G}\frac{1}{\left|G\right|}\sum_{u\in g}I_{\phi ,\varphi }\left({\vec{h}}_{\phi }^{u};H_{\phi }\left(G\right)\right)$$ (3)

where $I_{\phi ,\varphi }\left({\vec{h}}_{\phi }^{u};H_{\phi }\left(G\right)\right)$ is the Jensen-Shannon MI estimator, $\hat{\phi }$ is the parameter of the GIN defined in Equation 1, $\hat{\varphi }$ is the parameter used in the Jensen-Shannon mutual information estimator.

The objective function defined by Equation 3 can be used to optimize ${\hat{f}}_{g}$ defined in Equation 1. Additionally, it can be observed from Equations 1–3 that the optimization of ${\hat{f}}_{g}$ does not require sample labels, thus allowing the utilization of a large number of unlabeled samples for optimization.

In numerous application domains, features derived from pre-trained models often facilitate the direct execution of downstream tasks. However, within the realm of drug-target affinity prediction, directly applying features extracted from pre-trained models poses challenges. This stems from the fact that, in other domains, the pre-training samples align with those of the downstream task. Conversely, in drug-target affinity prediction, pre-training typically involves separate drug and target samples, whereas the prediction itself necessitates drug-target pair samples. This disparity in training samples hinders the ability of pre-trained models to comprehensively represent drug-target pairs. Consequently, pre-trained GINs are primarily employed to extract low-level features for drugs and targets, while the extraction of high-level drug-target pair features is entrusted to supervised training objectives. This complex learning objective is segmented into two stages, allowing for a more effective utilization of the strengths of each stage.

A target sequence typically consists of hundreds to thousands of amino acids. However, when this target interacts with a drug molecule to generate affinity, it is usually a specific amino acid fragment within the sequence that plays a major role. As a result, to better extract low-level features from the target sequence, the sequence is first divided into multiple fragments. The ideal method would be to segment the sequence based on the contribution of each fragment to affinity, but obviously, this is a more challenging task than affinity prediction itself. Fortunately, as this paper only utilizes pre-trained models to extract low-level features, a precise fragment segmentation method is not required. Therefore, a sliding window approach is adopted for fragment segmentation.

For the training data of the pre-trained target GIN $f_{\overset{˘}{G}}\left(\cdot \right)$ , a non-overlapping sliding window segmentation method is employed to prevent information leakage from overlapping sections during training. The Swiss-Prot dataset (Rafiei et al., 2024) is used for training. After applying the non-overlapping sliding window segmentation method to the targets on the Swiss-Prot dataset (Swiss-Port dataset), millions of target fragments can be obtained. After creating graphs for above fragments by using the RDkit tool (Landrum, 2016), the pre-trained target GIN $f_{G}\left(\cdot \right)$ can be trained by using the objective function defined in Equation 3.

After obtaining $f_{\overset{˘}{G}}\left(\cdot \right)$ , $f_{\overset{˘}{G}}\left(\cdot \right)$ can be used to extract low-level features of the target fragment. Given a target sequence $t_{j}=\left[t_{j,1},t_{j,2},\cdots ,t_{j,pj}\right]$ containing *p*_*i*_ amino acids. To extract low-level features, the target sequence tj needs to be first divided into fragments. Unlike the segmentation method used during the training of $f_{\overset{˘}{G}}\left(\cdot \right)$ , it employs an overlapping sliding window approach. This approach increases the probability of amino acid sequences that contribute to affinity being grouped into the same fragment. After segmenting the target sequence using overlapping sliding windows, the target graph features can be extracted as:

$$\overset{˘}{O}j=\left[f_{\overset{˘}{G}}\left(s_{j}^{1}\right),f_{\overset{˘}{G}}\left(s_{j}^{2}\right),\cdots ,f_{\overset{˘}{G}}\left(s_{j}^{K}\right)\right]$$ (4)

*K* represents the number of fragments the target is divided into.

It can be seen from Equation 4 that the graph features of all segments of tj are concatenated to obtain the target graph features of $t_{j}$ . Since $t_{j}$ is segmented using sliding windows, amino acid fragments that produce affinity may be distributed across several adjacent windows. Therefore, the output ${\overset{˘}{O}}_{j}$ will be further processed through a 2D CNN to obtain higher-level features that are more effective for predicting drug-target affinity.

A drug molecule typically consists of a few to several dozen atoms. When producing affinity with a target, most of the atoms in the drug contribute to this process. Therefore, the drug is not divided into multiple fragments but instead uses a pre-trained GIN to extract features for each atom of the drug, leveraging a large number of unlabeled training samples. The features that capture the influence of atoms and their relationships on affinity are further extracted by a supervised 2D CNN. For a large number of unlabeled training drugs, Equation 4 learns the pre-trained drug GIN $f_{\overset{⌢}{G}}\left(\cdot \right)$ . $f_{\overset{⌢}{G}}\left(\cdot \right)$ is used to extract atomic features from drug molecules. The CHEMBL dataset (Gaulton et al., 2017) is used for training $f_{\overset{⌢}{G}}\left(\cdot \right)$ .

Given a drug SMILES $d_{j}=\left[d_{j,1},d_{j,2},\cdots ,d_{j,pj}\right]$ containing *p*_*j*_ atoms, the drug node features ${\overset{⌢}{O}}_{j}$ can be extracted as following:

$$\overset{⌢}{O}j=f\overset{⌢}{G}\left(d_{j}\right)$$ (5)

Equation 4 shows that the low-level feature extraction results of targets are composed of fragments. Equation 5 shows that the low-level feature extraction results of drugs are composed of drug nodes. The pre- trained model is not directly used to extract the low-level features of the entire target and the entire drug, whose main reasons are as following. Firstly, Drug-target affinity is mainly determined by the local parts of drugs and targets. Using pre-training to extract the low-level features of the entire target and the entire drug will lose many features that are effective for DTA. Secondly, the pre-trained models in Equations 4, 5 are trained using a large number of unlabeled targets and unlabeled drugs, respectively, while DTAP uses drug-target sample pairs. In terms of training samples and training objectives, the pre-trained models and DTAP are too different. As a result, directly using the pre-trained entire low-level features of targets and drugs cannot meet the requirements of DTAP.

However, using Equations 4, 5 to extract the low-level features of drug targets, the pre-trained model only needs to achieve the training goal of maintaining the features of drugs and targets as much as possible. The features that are beneficial to DTAP only need to be handed over to the next step of extracting high-level features of drugs and targets. This is also the reason why our method performs excellently.

Algorithm 1. **Pseudo code of** pre-training target GIN *f_G_* ˘(.) and pre-training drug GIN *f_G_* ⌢(.).

**Input:** Pre-training drugs $\overset{⌢}{D}={\left[{\overset{⌢}{d}}_{1},{\overset{⌢}{d}}_{2},...,{\overset{⌢}{d}}_{n}\right]}^{T}$ , pre-training targets $\overset{˘}{T}={\left[{\overset{˘}{t}}_{1},{\overset{˘}{t}}_{2},\cdots ,{\overset{˘}{t}}_{m}\right]}^{T}$ , the length of the fragment of the target $\overset{˘}{l}$ .
**Output:** $f_{\overset{˘}{G}}\left(\cdot \right)$ and $f_{\overset{⌢}{G}}\left(\cdot \right)$ .
**Algorithm:**
**Stage 1: Training** $f_{\overset{˘}{G}}\left(\cdot \right)$

1: Divided fragments for $\overset{˘}{T}={\left[{\overset{˘}{t}}_{1},{\overset{˘}{t}}_{2},\cdots ,{\overset{˘}{t}}_{m}\right]}^{T}$ by non-overlapping sliding windows, where the window length is $\overset{˘}{l}$ .
2: Used the RDKit tool to construct graphs $\left[{\overset{˘}{g}}_{1},{\overset{˘}{g}}_{2},\cdots ,{\overset{˘}{g}}_{M}\right]\in \overset{˘}{G}$ for all target fragments
3: Used $\left[{\overset{˘}{g}}_{1},{\overset{˘}{g}}_{2},\cdots ,{\overset{˘}{g}}_{M}\right]\in \overset{˘}{G}$ to train target GIN $f_{\overset{˘}{G}}\left(\cdot \right)$ by the loss function that is defined by Equation 3.
**Stage 2: Training** $f_{\overset{⌢}{G}}\left(\cdot \right)$

4: Used RDkit tool to construct graphs $\left[{\overset{⌢}{g}}_{1},{\overset{⌢}{g}}_{2},\cdots ,{\overset{⌢}{g}}_{n}\right]\in \overset{⌢}{G}$ for all drugs $\overset{⌢}{D}={\left[{\overset{⌢}{d}}_{1},{\overset{⌢}{d}}_{2},...,{\overset{⌢}{d}}_{n}\right]}^{T}$ .
5: Used $\left[{\overset{˘}{g}}_{1},{\overset{˘}{g}}_{2},\cdots ,{\overset{˘}{g}}_{M}\right]\in \overset{˘}{G}$ to train target GIN $f_{\overset{˘}{G}}\left(\cdot \right)$ by the loss function that is defined by Equation 3.

Pre-trained Target GIN and Pre-trained Drug GIN mainly extract low-level features for targets and drugs. They are trained using large amounts of unlabeled target samples and drug samples based on the INFOGRAPH loss function defined in Equation 3. These features aim to preserve as much information about the targets and drugs themselves as possible, but they do not have the direct capability to predict drug-target affinity.

For drugs, the median lengths of SMILESs are approximately 46, 53, 48, 45, and 28 respectively on KIBA, Davis, DTC, Metz, and Tox-Cast. Therefore, after pre-training, zero-padding and truncation methods are adopted to fix the number of drug nodes extracted from Formula 5 at 64, which can not only retain the nodes of most drugs but also avoid introducing too many zero paddings. For targets, the median lengths of sequences are approximately 620, 632, 673, 631, and 479 respectively on KIBA, Davis, DTC, Metz, and Tox-Cast. Therefore, after pre-training, *K* in Formula 4 is set to 64, which can not only maintain an appropriate overlap of sliding windows but also make the low-level feature dimensions of drugs and targets the same. In particular, this can remove a large amount of redundant data, because the low-level features of the target are composed of fragment features.

### 2.4 High-level features extracted by CNN and DTA predicted by predictor

As can be seen from Equation 4, target graph features are composed of linked graph features of adjacent amino acid fragments, and drug-target affinity is mainly determined by a subset of amino acid fragments, which exhibits a distinct local receptive field, making it suitable for further feature extraction from target graph features using convolutional neural networks. Similarly, from Equation 5, drug node features are composed of linked node features, and drug-target affinity is also primarily determined by a subset of nodes, exhibiting a clear local receptive field, thus suitable for further feature extraction from drug node features using convolutional neural networks. Additionally, convolutional neural networks require fewer parameters to train, which is beneficial for addressing the relatively small number of drug-target pair samples.

Given $\overset{⌢}{O}i$ and ${\overset{˘}{O}}_{j}$ of a drug-target pair, the high- level features of $\overset{⌢}{O}i$ can be extracted by:

$$\overset{⌢}{P}i=\overset{⌢}{f}\overset{⌢}{C}\left({\overset{⌢}{O}}_{i}\right)$$ (6)

where ${\overset{⌢}{f}}_{\overset{⌢}{C}}\left(\cdot \right)$ is a shallow CNN that is used to extract high-level features of the target.

The high- level features of $\overset{⌢}{O}i$ can be extracted by:

$${\overset{˘}{P}}_{j}={\overset{˘}{f}}_{\overset{˘}{C}}\left({\overset{˘}{O}}_{j}\right)$$ (7)

where ${\overset{˘}{f}}_{\overset{˘}{C}}\left(\cdot \right)$ is a shallow CNN that is used to extract high-level features of the drug.

After extracting the high-level features of targets and drugs using Equations 6, 7, the drug-target pair features can be represented as:

$$P_{k}=\left[\overset{⌢}{P}i,{\overset{˘}{P}}_{j}\right]$$ (8)

After obtaining the drug-target pair features, a predictor can be used to predict the drug-target affinity. The predictor is defined as follows:

$$a_{k}=f_{P}\left(P_{k}\right)$$ (9)

where $f_{p}\left(\cdot \right)$ is a shallow predictor, which is consisted by many fully connected linear layers and active function.

${\overset{⌢}{f}}_{\overset{⌢}{C}}\left(\cdot \right)$ , ${\overset{˘}{f}}_{\overset{˘}{C}}\left(\cdot \right)$ and $f_{p\left(\cdot \right)}$ are trained by the mean-square error between the prediction score and actual score, which must be trained by the labeled drug-target affinity database.

### 2.5 The proposed GNPDTA model

GNPDTA is summarized in Algorithm 2, which comprises two stages: supervised GNPDTA training and GNPDTA testing. In the supervised GNPDTA training, the first six steps, introduced in Section 3.2, are used to extract low-level features for training samples. The final four steps, introduced in Section 3.3, are responsible for extracting high-level features for the training samples. During the GNPDTA testing, steps 11–16 are used to extract low-level features, while steps 17, 18, and 19 are utilized to extract high-level features and predict the DTA score for the test sample. The code is available at https://github.com/yeqing0713/GNPDTA.

Algorithm 2. Pseudo code of GNPDTA.

**Input:** **T**raining drugs $D={\left[d_{1},d_{2},...,d_{n}\right]}^{T}$ , training targets $T={\left[t_{1},t_{2},\cdots ,t_{m}\right]}^{T}$ , the DTA score *Y*, the length of the fragment of the target $\overset{˘}{l}$ , the number of fragments *S*, a test drug *d* and a test target *t*, the pre-trained target GIN $f_{\overset{˘}{G}}\left(\cdot \right)$ , the pre-trained drug GIN $f_{\overset{⌢}{G}}\left(\cdot \right)$ .
**Output:** the predicted DTA score a of *d* and *t*.
**Algorithm:**
**Stage 1: supervised GNPDTA training**
1: Divide target fragments for $T={\left[t_{1},t_{2},\cdots ,t_{m}\right]}^{T}$ by using overlapping sliding window method.
2: Used the RDkit tool to create graphs for all target fragments.
3: Extracted low-level features for targets by Equation 4.
4: Extracted high-level features for targets by Equation 6.
5: Used the RDkit tool to create graphs for $D={\left[d_{1},d_{2},...,d_{n}\right]}^{T}$ .
6: Extracted low-level features for drugs by Equation 5.
7: Extracted high-level features for drugs by Equation 7.
8: Calculated drug-target pair features by Equation 8.
9: Predicted drug-target affinity by Equation 9.
10: Trained ${\overset{⌢}{f}}_{\overset{⌢}{C}}\left(\cdot \right)$ , ${\overset{˘}{f}}_{\overset{˘}{C}}\left(\cdot \right)$ , $f_{p\left(\cdot \right)}$ by minimizing mean-square error between A and Y.
**Stage 2: GNPDTA testing**
11: Divided target fragments by using an overlapping sliding window method.
12: Used the RDkit tool to create graphs for all target fragments.
13: Extracted low-level features for *t* by Equation 4.
14: Extracted high-level features for *t* by Equation 6.
15: Used the RDkit tool to create graphs for d.
16: Extracted low-level features for *d* by Equation 5.
17: Extracted high-level features for *d* by Equation 7.
18: Calculated the drug-target pair features for *d* and *t* by Equation 9.
19: Predicted the DTA between *d* and *t* by Equation 9.

Compared with other existing DTAP methods, GNPDTA boasts the following advantage: GNPDTA employs a two-stage strategy for DTAP feature extraction. The first stage employs a self-supervised approach to learn low-level features, which can leverage a large amount of unlabeled training samples. This approach also helps overcome the large discrepancy between the self-supervised training objective and the drug-target affinity prediction objective. In the second stage, a supervised method is used to learn high-level features and predict DTA, enabling the utilization of limited samples to train the DTA predictor. It also allows for better utilization of the low-level features extracted from the self-supervised method. This two-stage methodology maximizes the strengths of both models, ensuring the optimal utilization of training samples. Notably, it minimizes the divide between self-supervised learning and DTA prediction, thereby enhancing the effectiveness of the features extracted through self-supervised learning.

### 2.6 Architectural parameter

GNPDTA comprises a pre-trained drug GIN, a pre-trained target GIN, two 2D CNNs, and a predictor. These neural networks involve numerous architectural parameters. The pre-trained drug GIN and the pre-trained target GIN primarily require setting the number of hidden layers and hidden size, which are set to 6 and 60 respectively, considering that the numbers of tokens in the drug and target are only 62 and 25. The 2D CNN needs to specify the number of hidden layers, the numbers of filters, and the kernel sizes. Specifically, the number of hidden layers is set to 3, with the numbers of filters for each layer being 32, 64, 128, and the kernel sizes being 5, 5, 3. As can be seen, this is a shallow 2D CNN. The reason for only using a shallow 2D CNN is that the current labeled drug-target affinity databases are still relatively small, making it difficult to support the training of neural networks with a large number of parameters. The Predictor also needs to define the number of hidden layers and hidden size, where the number of hidden layers is set to 3, and the hidden size for each layer is set to 512, 128, 1, because the scale of labeled training data of DTA is relatively small, which can only support the training of models with relatively few parameters. The training batch size is set to 1,280, which is the largest size for my GPU, where my GPU memory is 16 GB.

## 3 Results

### 3.1 Experimental setting

In this section, five datasets, Kiba, Davis, DTC, Metz, and Tox-Cast, listed in Table 1 are used to validate the proposed method. The same train/test splits as specified in (Wu et al., 2024) were adopted for the experiments, with 80% of the data instances used for training and 20% reserved for testing.

To evaluate the DTA predictions, the concordance index (CI) and mean squared error (MSE) were employed as metrics. Specifically, CI measures the ranking of the predicted binding affinity, as described in (Wu et al., 2024), while MSE quantifies the difference between the vector of predicted values and the vector of actual values, as stated in (Liao et al., 2022).

### 3.2 Ablation study and statistical test

The main contribution of GNPDTA lies in bridging the gap between pre-training and DTA objectives. By dedicating the first stage solely to low-level feature extraction, GNPDTA successfully narrows the gap between the pre-training objectives and the DTAP objectives. This approach ensures that the features learned in the pre-training phase are more relevant and useful for the downstream DTAP task.

Therefore, this subsection will compare the experimental results of the following methods: NO Pre-trained GIN+2D CNN, 2D CNN, and Pre-trained GIN+1D CNN. Among them, NO Pre-trained GIN+2D CNN has the same framework as GNPDTA, but the GIN model is not trained using large-scale unlabeled training data. The 2D CNN removes the GIN part from the framework and only uses 2D CNN to extract features of drugs and targets. Pre-trained GIN+1D CNN replaces the 2D CNN in the GNPDTA framework with 1D CNN. NO Pre-trained GIN+2D CNN can verify the effect of using pre-training. 2D CNN can validate the effect of using GIN to extract low-level features. Pre-trained GIN+1D CNN can verify the effect of using 2D CNN. The experimental results are presented in Tables 2–4. In order to test and compare the stability of algorithms, the mean and standard deviation of experimental results are given on the histogram.

**TABLE 2:** Results of DTA on Davis.

Method	Drug	Target	CI↑	MSE↓
DeepCDA	LSTM + 1D-CNN	LSTM + 1D-CNN	0.891 (0.003)	0.245
ELECTRA-DTA	ELECTRA + SE-CNN	ELECTRA + SE-CNN	0.889	0.238
MRBDTA	Transformer	Transformer	0.901	0.216
DeepGLSTM	GCN	LSTM	0.895	0.232
GCN-BERT	GCN	BERT	0.896	0.199
MGraphDTA	GCN	2D-CNN	0.900 (0.004)	0.207 (0.001)
DeepGS	GCN, BiGRU	2D-CNN	0.882	0.252
MFR-DTA	MLP	2D-CNN	0.905	0.221
MAM	2D-CNN	2D-CNN	0.891	0.183
WideDTA	1D-CNN	1D-CNN	0.886 (0.003)	0.262(0.009)
DeepDTA	1D-CNN	1D-CNN	0.878 (0.004)	0.261
MATT	1D-CNN	1D-CNN	0.891 (0.003)	0.227
AttentionDTA	1D-CNN	1D-CNN	0.887 (0.005)	0.262 (0.019)
SAG-DTA	GCN	1D-CNN	0.903	0.209
GraphDTA	GCN	1D-CNN	0.893	0.229
GSAML-DTA	GCN	GCN	0.896	0.201
GNPDTA	Pre-trained GIN + 2D-CNN	Pre-trained GIN + 2D-CNN	0.907 (0.004)	0.199 (0.003)

**TABLE 3:** Results of DTA on Kiba.

Method	Drug	Target	CI↑	MSE↓
DeepCDA	LSTM + 1D-CNN	LSTM + 1D-CNN	0.889 (0.002)	0.176
ELECTRA-DTA	ELECTRA + SE-CNN	ELECTRA + SE-CNN	0.889	0.162
MRBDTA	Transformer	Transformer	0.900	0.130
DeepGLSTM	GCN	LSTM	0.897	0.133
GCN-BERT	GCN	BERT	0.888	0.149
MGraphDTA	GCN	2D-CNN	0.902 (0.001)	0.128 (0.001)
DeepGS	GCN, BiGRU	2D-CNN	0.860	0.193
MFR-DTA	MLP	2D-CNN	0.898	0.136
MAM	2D-CNN	2D-CNN	0.898	0.135
WideDTA	1D-CNN	1D-CNN	0.875 (0.001)	0.179 (0.008)
DeepDTA	1D-CNN	1D-CNN	0.863 (0.002)	0.194
MATT	1D-CNN	1D-CNN	0.889 (0.004)	0.150
AttentionDTA	1D-CNN	1D-CNN	0.882 (0.004)	0.162 (0.003)
SAG-DTA	GCN	1D-CNN	0.892	0.130
GraphDTA	GCN	1D-CNN	0.891	0.139
GSAML-DTA	GCN	GCN	0.900	0.132
GNPDTA	Pre-trained GIN + 2D-CNN	Pre-trained GIN + 2D-CNN	0.906 (0.004)	0.126 (0.002)

**TABLE 4:** Results of DTA on DTC, Metz and Tox-cast.

Dataset	Method	Drug	Target	CI↑	MSE↓
DTC	DeepGLSTM	GCN	LSTM	0.895	0.149
	GraphDTA	GCN	1D-CNN	0.876	0.176
	GNPDTA	Pre-trained GIN + 2D-CNN	Pre-trained GIN + 2D-CNN	0.899	0.144
Metz	DeepGLSTM	GCN	LSTM	0.810	0.294
	GraphDTA	GCN	1D-CNN	0.800	0.317
	GNPDTA	Pre-trained GIN + 2D-CNN	Pre-trained GIN + 2D-CNN	0.812	0.283
Tox-cast	DeepGLSTM	GCN	LSTM	0.919	0.313
	GraphDTA	GCN	1D-CNN	0.915	0.324
	GNPDTA	Pre-trained GIN + 2D-CNN	Pre-trained GIN + 2D-CNN	0.921	0.301

**FIGURE 2:**
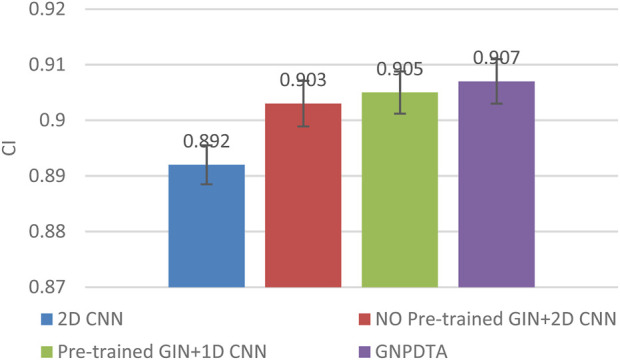
CI of DTA on Davis dataset based on ablation experiments.

**FIGURE 3:**
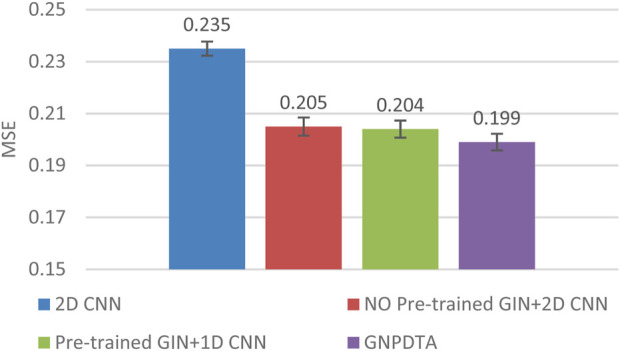
MSE of DTA on Davis dataset based on ablation experiments.

**FIGURE 4:**
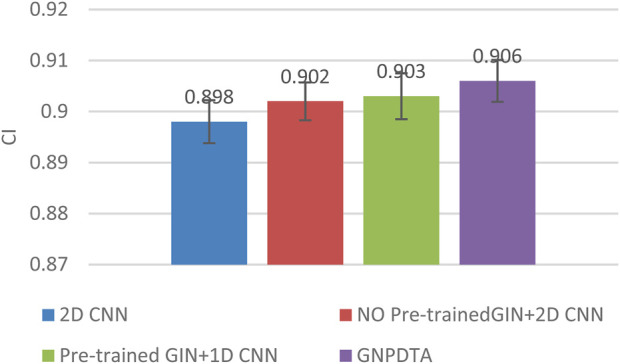
CI of DTA on Kiba dataset based on ablation experiments.

**FIGURE 5:**
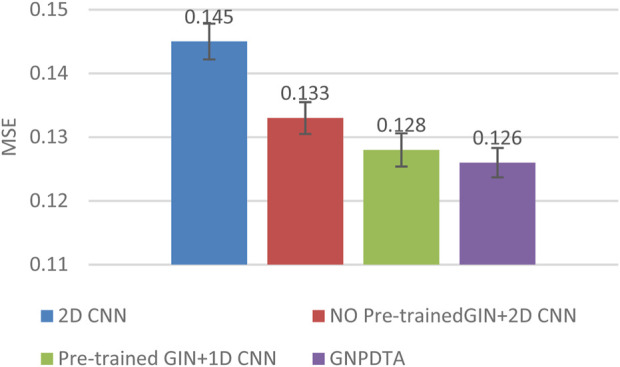
MSE of DTA on Kiba dataset based on ablation experiments.

From the mean values on Figures 2–5, it can be observed that GNPDTA’s CI is higher than 2D CNN’s CI by 0.015 and 0.008 on the Davis and Kiba datasets, respectively. Meanwhile, GNPDTA’s MSE is lower than 2D CNN’s MSE by 0.036 and 0.019 on the Davis and Kiba datasets, respectively. This indicates that the use of GIN to initially extract features is more effective than directly using one-hot features for improving the prediction performance of DTA. GNPDTA’s CI is also higher than NO Pre-trained GIN+2D CNN’s CI by 0.004 and 0.004 on the Davis and Kiba datasets, respectively, while GNPDTA’s MSE is lower by 0.006 and 0.007. This demonstrates that using a large number of unlabeled training samples helps improve the DTA prediction performance. Additionally, GNPDTA’s CI is higher than Pre-trained GIN+1D CNN’s CI by 0.002 and 0.003 on the Davis and Kiba datasets, respectively, while GNPDTA’s MSE is lower than 2D CNN’s MSE by 0.005 and 0.002. This suggests that 2D CNN is better able to comprehensively utilize the primary features extracted by Pre-trained GIN. The reason is that 2D CNN not only extracts features between sequences but also combines existing features to create new ones.

From the variance on Figure 2, the standard deviations of CIs of the four methods on the Davis dataset are respectively 0.003, 0.004, 0.004, and 0.004. From the variance on Figure 3, the variances of MSEs of the four methods on the Davis dataset are respectively 0.003, 0.004, 0.003, and 0.003. From the variance on Figure 4, the variances of CIs of the four methods on the Kiba dataset are respectively 0.004, 0.004, 0.005, and 0.004. From the variance on Figure 5, the variances of MSEs of the four methods on the Kiba dataset are respectively 0.003, 0.003, 0.003, and 0.002. These data indicate that our method’s experimental results are relatively stable and have strong practical application capabilities.

### 3.3 Results of DTA on davis

In this subsection, GNPDTA is compared with numerous recent studies on Davis, including DeepDTA (Öztürk et al., 2018), MATT (Zeng et al., 2021), WideDTA (Öztürk et al., 2019), DeepCDA (Karim et al., 2020), ELECTRA-DTA (Wang et al., 2022), MAM (Aleb, 2021), MRBDTA (Zeng et al., 2023), AttentionDTA (Zhao et al., 2019), SAG-DTA (Zhang et al., 2021), GraphDTA (Nguyen et al., 2021), MGraphDTA (Yang et al., 2022), DeepGS (Lin, 2020), DeepGLSTM (Mukherjee et al., 2022), GCN-BERT (Lennox et al., 2021), GSAML-DTA (Nguyen et al., 2021), and MFR-DTA (Hu et al., 2023).

According to the principles of the feature extraction of methods, they can be broadly classified into several categories. Two-stage-based methods include DeepCDA and ELECTRA-DTA. Pre-training-based methods comprise DeepCDA, ELECTRA-DTA, MRBDTA, DeepGLSTM, and GCN-BERT. 2D-CNN-based methods are MGraphDTA, DeepGS, MFR-DTA, and MAM. 1D-CNN-based methods are WideDTA, DeepDTA, MATT, AttentionDTA, SAG-DTA, and GraphDTA. GCN-based methods cover DeepGLSTM, GCN-BERT, MGraphDTA, DeepGS, SAG-DTA, GraphDTA, GSAML-DTA, and DGraphDTA. The model evaluations of the DTA on Davis are listed in Table 2, where the best of each group on each dataset is shown in the bold font, “↑” represents that the larger the value in this column is, the better it is, “↓” represents that the smaller the value in this column is, the better it is. The value outside the parentheses is the mean, and the value inside the parentheses is the standard deviation. It can be seen from Table 2 that GNPDTA is the best among all compared methods on Davis.

Firstly, GNPDTA significantly outperforms two-stage-based methods. Specifically, the CI of GNPDTA is 0.016 and 0.018 higher, respectively, compared to that of DeepCDA and ELECTRA-DTA. Similarly, the MSE of GNPDTA is 0.046, and 0.039 lower, respectively, than that of DeepCDA and ELECTRA-DTA. Notably, the CI of GNPDTA is 0.016 higher than the CI of DeepCDA, which is the best-performing method among two-stage-based methods. Moreover, the MSE of GNPDTA is 0.046 lower than the MSE of DeepCDA. These results demonstrate that our method is more effective than the compared two-stage-based methods for DTAP.

Secondly, GNPDTA significantly outperforms pre-training-based methods. Specifically, the CI of GNPDTA is 0.016, 0.018, 0.006, 0.012, and 0.011 higher, respectively, compared to that of DeepCDA, ELECTRA-DTA, MRBDTA, DeepGLSTM and GCN-BERT. Similarly, the MSE of GNPDTA is 0.046, 0.039, 0.017, 0.033, and 0.0 lower, respectively, than that of DeepCDA, ELECTRA-DTA, MRBDTA, DeepGLSTM and GCN-BERT. Notably, the CI of GNPDTA is 0.0061 higher than the CI of MRBDTA, which is the best-performing method among CNN-based approaches. Moreover, the MSE of GNPDTA is 0.017 lower than the MSE of MRBDTA. These results demonstrate that our method is more effective than the compared two-stage-based methods for DTAP.

Thirdly, it is evident that GNPDTA surpasses 2D-CNN-based methods in terms of performance. Specifically, GNPDTA’s CI surpasses MGraphDTA, DeepGS, MFR-DTA, and MAM by 0.007, 0.025, 0.002 and 0.016, respectively. Similarly, its mean squared error (MSE) is lower, specifically 0.008, 0.053, 0.022 and −0.016 less, compared to MGraphDTA, DeepGS, MFR-DTA, and MAM. Notably, GNPDTA’s CI exceeds the CI of MFR-DTA, the top-performing CNN-based approach, by 0.007. Furthermore, GNPDTA’s MSE is 0.008 lower than MFR-DTA’s MSE. These findings clearly indicate that our proposed method exhibits superior efficacy compared to the examined two-stage-based methods for DTAP.

Fourthly, it is evident that GNPDTA surpasses 1D-CNN-based methods in terms of performance. Specifically, GNPDTA’s CI surpasses WideDTA, DeepDTA, MATT, AttentionDTA, SAG-DTA and GraphDTA by 0.021, 0.029, 0.016, 0.02, 0.004 and 0.014, respectively. Similarly, its mean squared error (MSE) is lower, specifically 0.063, 0.062, 0.028, 0.063, 0.01 and 0.03 less, compared to WideDTA, DeepDTA, MATT, AttentionDTA, SAG-DTA and GraphDTA. GNPDTA’s CI exceeds the CI of SAG-DTA, the top-performing CNN-based approach, by 0.004. Furthermore, GNPDTA’s MSE is 0.01 lower than SAG-DTA’s MSE. These findings clearly indicate that our proposed method exhibits superior efficacy compared to the examined two-stage-based methods for DTAP.

Fifthly, it is evident that GNPDTA surpasses 2D-CNN-based methods in terms of performance. Specifically, GNPDTA’s CI surpasses DeepGLSTM, GCN-BERT, MGraphDTA, DeepGS, SAG-DTA, GraphDTA, GSAML-DTA, DGraphDTA by 0.012, 0.011, 0.007, 0.025, 0.004, 0.014, 0.011 and 0.003, respectively. Similarly, its mean squared error (MSE) is lower, specifically 0.033, 0, 0.008, 0.053, 0.01, 0.03, 0.002 and 0.003 less, compared to DeepGLSTM, GCN-BERT, MGraphDTA, DeepGS, SAG-DTA, GraphDTA and GSAML-DTA. Notably, GNPDTA’s CI exceeds the CI of GSAML-DTA, the top-performing CNN-based approach, by 0.003. Furthermore, GNPDTA’s MSE is 0.003 lower than GSAML-DTA’s MSE. These findings clearly indicate that our proposed method exhibits superior efficacy compared to the examined two-stage-based methods for DTAP.

### 3.4 Results of DTA on Kiba

In this subsection, GNPDTA is also compared with numerous recent studies on Davis, including DeepDTA (Öztürk et al., 2018), MATT (Zeng et al., 2021), WideDTA (Öztürk et al., 2019), DeepCDA (Karim et al., 2020), ELECTRA-DTA (Wang et al., 2022), MAM (Aleb, 2021), MRBDTA (Zeng et al., 2023), AttentionDTA (Zhao et al., 2019), SAG-DTA (Hu et al., 2023), GraphDTA (Nguyen et al., 2021), MGraphDTA (Yang et al., 2022), DeepGS (Lin, 2020), DGraphDTA (Lu et al., 2020), DeepGLSTM (Mukherjee et al., 2022), GCN-BERT (Lennox et al., 2021), GSAML-DTA (Pan et al., 2023), and MFR-DTA (Zhang Xianfeng et al., 2023). The model evaluations of the DTA on Kiba are listed in Table 3, where the best of each group on each dataset is shown in the bold font, “↑” represents that the larger the value in this column is, the better it is, “↓” represents that the smaller the value in this column is, the better it is. It can be seen from Table 3 that GNPDTA is the best among all compared methods on Kiba.

Firstly, GNPDTA significantly outperforms two-stage-based methods. Specifically, the CI of GNPDTA is 0.017 and 0.017 higher, respectively, compared to that of DeepCDA and ELECTRA-DTA. Similarly, the MSE of GNPDTA is 0.05 and 0.036 lower, respectively, than that of DeepCDA and ELECTRA-DTA. Notably, the CI of GNPDTA is 0.017 higher than the CI of ELECTRA-DTA, which is the best-performing method among two-stage-based methods. Moreover, the MSE of GNPDTA is 0.036 lower than the MSE of ELECTRA-DTA. These results demonstrate that our method is more effective than the compared two-stage-based methods for DTAP.

Secondly, GNPDTA significantly outperforms pre-training-based methods. Specifically, the CI of GNPDTA is 0.017, 0.017, 0.006, 0.009, and 0.018 higher, respectively, compared to that of DeepCDA, ELECTRA-DTA, MRBDTA, DeepGLSTM and GCN-BERT. Similarly, the MSE of GNPDTA is 0.05, 0.036, 0.004, 0.007 and 0.023 lower, respectively, than that of DeepCDA, ELECTRA-DTA, MRBDTA, DeepGLSTM and GCN-BERT. Notably, the CI of GNPDTA is 0.006 higher than the CI of MRBDTA, which is the best-performing method among CNN-based approaches. Moreover, the MSE of GNPDTA is 0.004 lower than the MSE of MRBDTA. These results demonstrate that our method is more effective than the compared two-stage-based methods.

Thirdly, it is evident that GNPDTA surpasses 2D-CNN-based methods in terms of performance. Specifically, GNPDTA’s CI surpasses MGraphDTA, DeepGS, MFR-DTA, and MAM by 0.004, 0.046, 0.008 and 0.008, respectively. Similarly, its mean squared error (MSE) is lower, specifically 0.002, 0.067, 0.01 and −0.009 less, compared to MGraphDTA, DeepGS, MFR-DTA, and MAM. Notably, GNPDTA’s CI exceeds the CI of MGraphDTA, the top-performing CNN-based approach, by 0.004. Furthermore, GNPDTA’s MSE is 0.002 lower than MGraphDTA’s MSE. They clearly indicate that our proposed method exhibits superior efficacy compared to the examined two-stage-based methods for DTAP.

Fourthly, it is evident that GNPDTA surpasses 1D-CNN-based methods in terms of performance. Specifically, GNPDTA’s CI surpasses WideDTA, DeepDTA, MATT, AttentionDTA, SAG-DTA and GraphDTA by 0.031, 0.043, 0.017, 0.024, 0.014 and 0.015, respectively. Similarly, its mean squared error (MSE) is lower, specifically 0.053, 0.068, 0.024, 0.036, 0.004 and 0.013 less, compared to WideDTA, DeepDTA, MATT, AttentionDTA, SAG-DTA and GraphDTA. GNPDTA’s CI exceeds the CI of SAG-DTA, the top-performing CNN-based approach, by 0.014. Furthermore, GNPDTA’s MSE is 0.004 lower than SAG-DTA’s MSE. These findings clearly indicate that our proposed method exhibits superior efficacy compared to the examined two-stage-based methods for DTAP.

Fifthly, it is evident that GNPDTA surpasses 2D-CNN-based methods in terms of performance. Specifically, GNPDTA’s CI surpasses DeepGLSTM, GCN-BERT, MGraphDTA, DeepGS, SAG-DTA, GraphDTA, GSAML-DTA, DGraphDTA by 0.009, 0.018, 0.004, 0.046, 0.014, 0.015 and 0.006, respectively. Similarly, its mean squared error (MSE) is lower, specifically 0.007, 0.023, 0.002, 0.067, 0.004, 0.013 and 0.006 less, compared to DeepGLSTM, GCN-BERT, MGraphDTA, DeepGS, SAG-DTA, GraphDTA and GSAML-DTA. Notably, GNPDTA’s CI exceeds the CI of GSAML-DTA, the top-performing CNN-based approach, by 0.004. Furthermore, GNPDTA’s MSE is 0.002 lower than GSAML-DTA’s MSE. These findings clearly indicate that our proposed method exhibits superior efficacy compared to the examined two-stage-based methods for DTAP.

### 3.5 Results of DTA on DTC, Metz and Tox-cast

In this subsection, GNPDTA is compared against GraphDTA and DeepGLSTM on the DTC, Metz, and Tox-cast datasets. However, as only a limited number of deep learning methods have been validated on these three databases, our comparison is primarily focused on GraphDTA and DeepGLSTM. The resulting comparisons are presented in Table 4, where the best of each group on each dataset is shown in the bold font, “↑” represents that the larger the value in this column is, the better it is, “↓” represents that the smaller the value in this column is, the better it is. It can be seen from Table 2 that GNPDTA is the best among all compared methods on Davis.

From Table 4, it is evident that our proposed method outperforms others. Firstly, the CI of GNPDTA is higher than DeepGLSTM by 0.004, 0.002, and 0.002, respectively, while the MSE of GNPDTA is lower by 0.005, 0.011, and 0.012, respectively, on the DTC, Metz, and Tox-Cast datasets. Secondly, when comparing GNPDTA with GraphDTA on the same datasets, GNPDTA’s CI is higher by 0.023, 0.012, and 0.006, while its MSE is lower by 0.032, 0.034, and 0.023, respectively. These results demonstrate that combining pre-trained GIN with 2D CNN to learn high-level features for drugs and targets is beneficial for drug-target affinity prediction.

## 4 Discussion

In this paper, we introduce GNPDTA as a novel approach for predicting DTA, with the aim of addressing the significant discrepancy between the pre-training objectives and samples utilized in existing pre-training methods and their corresponding DTAP methods. GNPDTA integrates a two-stage strategy for feature extraction in the context of DTAP. Initially, a self-supervised learning mechanism is employed to acquire low-level features from unlabeled data, thereby bridging the gap between the objectives of self-supervised learning and DTA prediction, as well as the discrepancy in samples used for both purposes. Subsequently, a supervised approach is leveraged to refine high-level features and predict DTA using limited labeled samples, effectively incorporating the low-level features obtained from the first stage. This two-stage methodology maximizes the strengths of both models, ensuring the optimal utilization of training samples. Notably, it minimizes the divide between self-supervised learning and DTA prediction, thereby enhancing the effectiveness of the features extracted through self-supervised learning. Our findings demonstrate that GNPDTA surpasses existing methods, indicating its potential for more efficient applications in DTAP.

GNPDTA offers valuable insights into the complex interactions between drugs and their targets. The ability to accurately predict DTA has profound implications for drug discovery and development. By identifying potential drug-target pairs with high affinity, researchers can prioritize compounds for further experimentation, thereby accelerating the drug development process. Moreover, the self-supervised learning component of GNPDTA captures information beyond direct DTA annotations, potentially uncovering novel patterns and relationships within the drug-target interaction landscape. The practical implications of GNPDTA are significant. In the pharmaceutical industry, the ability to accurately predict DTA could revolutionize drug screening and optimization, reducing costs and time-to-market. Furthermore, GNPDTA has the potential to facilitate the discovery of novel drug indications by predicting off-target effects and repurposing existing drugs for new therapeutic applications. Additionally, in the context of precision medicine, GNPDTA could aid in the selection of personalized treatment options by predicting an individual’s response to various drugs based on their unique genetic profile.

There are several interesting problems to be investigated in our future work. As one of the further works, optimized and improved the GNPDTA model by exploring more advanced graph neural network structures and more efficient feature fusion methods, which aims to further enhance the accuracy and efficiency of drug-target affinity prediction. As another further work, investigated and developed visualization techniques for the model, which can improve the interpretability of DTAP.

## Data Availability

The original contributions presented in the study are included in the article/supplementary material, further inquiries can be directed to the corresponding author.
